# Thioredoxin system modulates metabolic and stomatal responses to elevated CO_2_ in Arabidopsis

**DOI:** 10.1111/nph.71302

**Published:** 2026-05-29

**Authors:** Paula da Fonseca‐Pereira, Domingos F. Mélo Neto, Rita de Cássia Monteiro‐Batista, Daniel Gomes Coelho, Jaciara Lana‐Costa, Leonardo Perez de Souza, Ina Krahnert, Danilo M. Daloso, Jorge Gago, Alisdair R. Fernie, Wagner L. Araújo, Adriano Nunes‐Nesi

**Affiliations:** ^1^ National Institute of Science and Technology on Plant Physiology under Stress Conditions, Departamento de Biologia Vegetal Universidade Federal de Viçosa Viçosa 36570‐900 Minas Gerais Brazil; ^2^ Max‐Planck‐Institute of Molecular Plant Physiology Am Mühlenberg 1 Potsdam‐Golm 14476 Germany; ^3^ Departamento de Bioquímica e Biologia Molecular Universidade Federal do Ceará Fortaleza 60451‐970 Ceará Brazil; ^4^ Agro‐Environmental and Water Economics Institute (INAGEA), Research Group of Plant Biology Under Mediterranean Conditions, Department of Biology Universitat de Les Illes Balears Palma 07122 Illes Balears Spain

**Keywords:** *Arabidopsis thaliana*, elevated CO_2_, plant acclimation, redox homeostasis, stomatal dynamics thioredoxin system

## Abstract

- The NADPH‐dependent thioredoxin reductase/thioredoxin (NTR/TRX) system plays a central role in maintaining redox homeostasis of the cell by transferring electrons from NADPH to target proteins though NTR and TRX, thereby modulating cysteine redox states and regulating enzyme activity. However, the specific contribution of the extraplastidial NTR/TRX system to plant acclimation to elevated CO_2_ (eCO_2_) remains poorly understood.
- Here, we investigated the physiological and metabolic responses of Arabidopsis mutants deficient in mitochondrial TRXo1 (*trxo1*) or in the cytosolic/mitochondrial/nuclear thioredoxin reductases NTRA/NTRB (*ntrantrb*) alongside the wild‐type (WT), grown under ambient (aCO_2_; 400 ppm) and eCO_2_ (800 ppm) CO_2_ conditions.
- The stomatal closure induced by abscisic acid or eCO_2_ was partially compromised in *ntrantrb* double mutant. The stomatal density decreased in WT and *ntrantrb* plants under eCO_2_, while did not change in *trxo1* lines. The mutants showed much higher increases in rosette biomass under eCO_2_ compared to WT. This was associated with alterations in both primary and secondary metabolisms, but not to the level of NAD(P)(H), and reduced glutathione/oxidized glutathione (GSH : GSSG) ratio.
- Our results indicate that TRXo1 and NTRA/B play key roles in regulating stomatal development/movement and both primary and secondary metabolisms, thereby impacting plant acclimation to eCO_2_.

## Graphical abstract

Integrative model of extraplastidial NADPH‐dependent thioredoxin reductase/thioredoxin system‐mediated regulation of stomatal function, redox homeostasis, and metabolic responses under ambient and elevated CO_2_ in *Arabidopsis thaliana*.

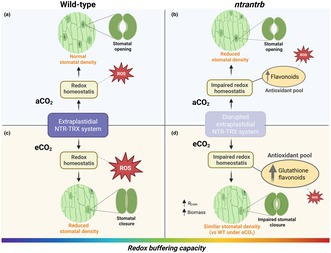

## Introduction

Redox regulation is essential for maintaining cellular homeostasis and coordinating physiological processes in plants, including primary metabolism, oxidative stress responses, and environmental acclimation (Cejudo *et al*., 2021; Liu *et al*., 2024; Mittler & Jones, 2024). Thioredoxins (TRXs), a family of thiol‐oxidoreductases characterized by a conserved Cys‐X‐X‐Cys motif, function by modulating the redox state of target proteins (Meyer *et al*., 2021). In *Arabidopsis thaliana* L., > 20 TRX isoforms have been identified and classified according to sequence similarity and subcellular localization (Belin *et al*., 2015; Geigenberger *et al*., 2017). In chloroplasts, TRXs play a well‐established role in regulating enzymes related to starch metabolism, antioxidant responses, and Calvin–Benson–Bassham cycle (Michelet *et al*., 2013; Kapoor *et al*., 2015; Okegawa & Motohashi, 2015). Additionally, TRXs also participate in mitochondrial redox control (Yoshida *et al*., 2013; Martí *et al*., 2020).

Proteomic studies have identified over 100 putative mitochondrial TRX targets (Yoshida *et al*., 2013). Within mitochondria, TRXs modulate key enzymes involved in photorespiration and central carbon metabolism, including components of the tricarboxylic acid (TCA) cycle, the glycine decarboxylase complex (GDC), and the alternative oxidase (AOX) (Daloso *et al*., 2015; Reinholdt *et al*., 2019; Da Fonseca‐Pereira *et al*., 2021). While plastidial TRXs are reduced by ferredoxin‐thioredoxin reductase or NADPH‐dependent thioredoxin reductase C (NTRC) (Thormählen *et al*., 2015), mitochondrial TRXo, and cytosolic TRXh isoforms mostly depend on the NADPH‐dependent thioredoxin reductases NTRA and NTRB for their reduction (Marchal *et al*., 2014). The NTR/TRX system plays a crucial role in maintaining redox homeostasis, supporting stress adaptation and energy metabolism, particularly under fluctuating environmental conditions such as variations in atmospheric CO_2_ levels (Mhamdi & Noctor, 2016; Souza *et al*., 2023).

Elevated atmospheric CO_2_ (eCO_2_) is expected to impact plant metabolism and development in the coming decades (Gamage *et al*., 2018). eCO_2_ reduces photorespiration and enhances photosynthetic carbon fixation, leading to shifts in cellular metabolism that require tight coordination between chloroplasts and mitochondria. Such changes impose constraints on redox homeostasis, requiring tight coordination between electron transport, reactive oxygen species (ROS) production, and antioxidant defenses must be finely tuned. Within mitochondria, the NTR/TRX system contributes to the redox regulation of key components such as AOX, which helps balance electron flow and limit ROS formation during stress (Florez‐Sarasa *et al*., 2019; Umekawa & Ito, 2019). Additionally, the NTR/TRX system modulates enzymes of the TCA cycle: It activates citrate synthase 4 and NAD^+^‐dependent isocitrate dehydrogenase through TRX‐mediated reduction, while inactivating succinate dehydrogenase and mitochondrial fumarase via thiol switching (Schmidtmann *et al*., 2014; Yoshida & Hisabori, 2014; Daloso *et al*., 2015). These dynamic redox regulations ensure that mitochondrial metabolism adapts to the changing demands imposed by eCO_2_, maintaining coordination with photosynthetic processes and stomatal function (Da Fonseca‐Pereira *et al*., 2021; Timm *et al*., 2024).

The shift in the CO_2_/O_2_ balance under eCO_2_ increases the carboxylation efficiency of ribulose‐1,5‐bisphosphate carboxylase/oxygenase, enhancing carbon fixation. This shift influences redox homeostasis in chloroplasts and mitochondria, creating a demand for coordinated adjustments to maintain metabolic stability (Foyer & Noctor, 2020). Adjustments in redox metabolism under eCO_2_ have been linked to improvements in water‐use efficiency (WUE), as altered ROS dynamics – play central roles in the signaling pathways that regulate stomatal function and, consequently, WUE (AbdElgawad *et al*., 2015). Recently, De Brasi‐Velasco *et al*. (2023) demonstrated that TRX o1 contributes to abscisic acid (ABA) perception by modulating cellular redox balance and downstream signaling cascades. This redox regulation intersects ABA‐, ROS‐, and calcium‐dependent pathways that coordinate stomatal closure (Liu *et al*., 2022).

The interplay among ROS, thiol‐based redox signaling, and genes associated with redox homeostasis further influences stomatal behavior and plant development (Sevilla *et al*., 2023). Plastidial and cytosolic thiol reductases, including TRXs, glutaredoxins (GRXs), and peroxiredoxins (PRXs), play pivotal roles in the redox regulation of guard cell proteins, directly impacting stomatal functioning (Montillet *et al*., 2021). Functional studies have demonstrated that mutants deficient in NADPH‐dependent TRXs or plastidial 2‐CysPRXs exhibit altered stomatal conductance, leaf temperature, and ABA‐ or ROS‐induced stomatal closure, highlighting the importance of redox switches in stomatal signaling (Montillet *et al*., 2021). Moreover, mitochondrial TRXo1 also regulates stomatal aperture and development, likely through its interaction with ROS, ABA, and nitric oxide signaling, without significantly affecting photosynthetic performance (Sánchez‐Guerrero *et al*., 2021). Given the pivotal role of stomatal function in WUE and plant productivity, elucidating how TRXs mediate redox regulation under eCO_2_ conditions is crucial for anticipating plant adaptation to future atmospheric CO_2_ scenarios (Wang *et al*., 2019).

Given the central role of redox regulation in plant physiology, we hypothesized that the NTR/TRX system may directly influence stomatal responses to CO_2_ by modulating the redox state of key signaling components and/or metabolites that integrate photosynthesis and stomatal conductance, such as sugars and organic acids. This regulatory network likely links metabolic adjustments to gas exchange processes, ultimately affecting carbon assimilation. However, the specific contributions of mitochondrial TRXs and NTRs to plant responses under eCO_2_ remain unclear. Here, we investigated the roles of *TRXo1*, *NTRA*, and *NTRB* genes in the metabolic and physiological responses to eCO_2_, with particular focus on stomatal development and conductance. To address this, we performed an extensive metabolic characterization, including primary and secondary metabolism, as well as the level of starch, NAD(P)(H), and GSH/GSSG contents, coupled with stomatal morphological and gas exchange analyses in Arabidopsis *trxo1* and *ntrantrb* mutants and the wild‐type (WT) (Col‐0) grown under aCO_2_ and eCO_2_ conditions. Our findings provide new insights into the contribution of the NTRA/B‐TRXo1 system to redox regulation and metabolic plasticity, underscoring its broader implications for plant acclimation to future atmospheric CO_2_ scenarios.

## Materials and Methods

### Plant material and growth conditions

All *Arabidopsis thaliana* (L.) Heynh. plants used in this work were of the Columbia (Col‐0) ecotype. All mutant lines *ntrantrb*, *trxo1‐1* (SALK 042792) and *trxo1‐2* (SALK_143294) have been previously characterized (Reichheld *et al*., 2007). The seeds of the four genotypes were sown on standard glasshouse soil (Stender substrate) in plastic pots with a 0.1 l capacity. Trays containing the pots were maintained under a 12 h : 12 h, light : dark period (22°C : 16°C) with relative humidity of 60/75% and a light intensity of 150 μmol photons m^−2^ s^−1^. Fourteen days after sowing, plants were transferred individually to new pots (0.1 l). Three days after the transfer (17 d after sowing), pots were randomly arranged in trays and placed in controlled‐environment growth chambers under the same temperature, humidity and light conditions as mentioned previously, except for the CO_2_ concentration, which was maintained at either ambient (aCO_2_; 400 μmol mol^−1^) or elevated (eCO_2_; 800 μmol mol^−1^) levels. For all subsequent analyses, a minimum number of six biological replicates were used.

### Gas exchange measurements and stomatal conductance dynamics

Gas exchange measurements were conducted on 28‐d‐old plants sampled during the middle of the light period. They were determined using an open‐flow infrared gas exchange analyzer system (LI‐6400XT; LI‐COR Biosciences, Lincoln, NE, USA) equipped with an integrated fluorescence chamber (LI‐6400‐40; LI‐COR Biosciences). Point measurements were taken using a 2‐cm^−2^ leaf chamber, artificial light at an intensity of 150 μmol photons m^−2^ s^−1^ with 10% blue light, reference CO_2_ concentration of 400 ppm, vapor pressure deficit (VPD) between 1 and 1.3 Kpa, and block temperature 25°C. To analyze plant growth under high [CO_2_], the reference [CO_2_] was adjusted to 800 ppm. In the analysis of *g*_S_ dynamics as a function of CO_2_ concentration, *g*_S_ measurements were taken at 1 min intervals. For responses to CO_2_ concentration transitions, leaves were exposed to 400/800/400 μmol CO_2_ mol^−1^ air for 20/40/40 min, and all light, VPD, and block temperature settings were the same as those used in point measurements.

### Stomatal opening assay on detached leaves

Stomatal opening assays were performed on detached leaves followed the procedure described by Araújo *et al*. (2011). The fully expanded fifth leaf of 5‐wk‐old plants was detached at least 2 h after the onset illumination and floated on stomatal opening buffer containing 10 mM KCl, 50 μM CaCl_2_, and 5 mM MES‐Tris (pH 6.15) for 2 h under light (150 μmol photons m^−2^ s^−1^) to promote stomatal opening. After this pre‐incubation, ethanol (as a solvent control) or ABA (final concentration 10 μM, diluted in 0.1% ethanol) was added to the buffer, and the leaves were incubated for an additional 2 h under the same light condition. Following treatment, leaves were gently dried, and the adaxial surface was affixed to transparent adhesive tape. The epidermis was carefully peeled and immediately image using an optical microscope (Zeiss AX10) equipped with a digital camera (Axiocam MRc). Stomatal aperture was measured manually using the ImageJ software (v.1.42q; NIH USA, http://rsbweb.nih.gov/ij/). Four leaves from independent plants were analyzed per genotype, and at least 15 stomata per leaf were measured, totalizing a minimum of least 60 stomata per genotype.

### Stomatal density, index and size

Leaves from 28‐d‐old plants grown under aCO_2_ and eCO_2_ conditions were collected in tubes containing methanol. Leaves were subsequently transferred to new tubes containing lactic acid and heated at 95°C until complete diaphanization. Images of the abaxial epidermis were captured using an optical microscope (Zeiss; model AX10) coupled with a digital camera (AxiocamMRc). Leaves from six plants per genotype were placed on microscope slides containing a drop of water, and eight areas of the abaxial surface of each leaf were photographed and analyzed using ImageJ (Schindelin *et al*., 2015). The stomatal index was determined by the ratio between the number of stomata and the total number of epidermal cells (stomata and pavement cells), multiplied by 100, referring to the proportion of stomatal relative to the total epidermal cell number.

### Soluble protein totals, starch, chls, and metabolite profile

Total soluble proteins, starch, chls, and metabolite contents were determined from whole rosettes. Samples were harvested in the middle of the light period, immediately frozen in liquid nitrogen, and stored at −80°C until extraction. Metabolite extraction was performed as described by Fernie *et al*. (2002). Approximately 30 mg of fresh and macerated tissue was mixed with 700 μl of methanol, vortexed, and incubated at 80°C for 20 min under shaking. After centrifugation (9500 ***g***, 10 min, 4°C), the supernatant was collected, and the pellet was used for the determination of total soluble proteins (Bradford, 1976) and starch (Fernie *et al*., 2002). A portion of the supernatant was used for Chl quantification following Porra *et al*. (1989), while the remaining extract was subjected to liquid–liquid partitioning with chloroform. The methanol/water phase was used for profiling of primary and secondary metabolites via GC‐MS and LC‐MS, respectively (Lisec *et al*., 2006). Ribitol and isovitexin were included as internal standards in the GC‐MS and LC‐MS analyses, respectively.

Chromatograms and mass spectra were processed using Chroma TOF 1.0 (LECO Corporation, St. Joseph, MI, USA, http://www.leco.com/) and TagFinder 4.0 (Luedemann *et al*., 2008) software. Metabolites identification was based on comparison with authentic standards and reference spectra from the Golm Metabolome Database (Kopka *et al*., 2005).

### Glutathione and nicotinamide adenine dinucleotide contents

The contents of reduced (GSH) and oxidized (GSSG) glutathione were determined as described by Foyer *et al*. (1991) and Rahman *et al*. (2007). Pyridine nucleotides assays followed the procedure of Queval & Noctor (2007), with is based on the selective hydrolysis of the reduced forms (NADH and NADPH) in acidic medium and of the oxidized forms (NAD^+^ and NADP^+^) in alkaline medium. Quantification was performed using the phenazine methosulfate‐catalyzed reduction of dichlorophenolindophenol (DCPIP) in the presence of ethanol and alcohol dehydrogenase (for NAD^+^ and NADH) or glucose 6‐phosphate (G6P) and G6P dehydrogenase (for NADP^+^ and NADPH).

### Statistical analysis

The data were initially submitted to the Shapiro–Wilk normality test and then to the ANOVA and Tukey tests, at 5% probability. For the data of the first experiment, we considered an experiment with single‐factor (genotypes) in completely randomized design, while for the second experiment, we considered a double factorial experiment (genotypes and [CO_2_]) also in completely randomized design. In cases where the interaction was not significant at 5% probability, the analysis of isolated factors was performed. The results were expressed using the mean and SE, with figures plotted using Microsoft Excel® (2021), and statistical analyses were performed using Rbio software (Bhering, 2017). For the metabolite profile data, the raw abundances of the ions were initially subtracted from the blank sample and standardized by the internal standard and initial fresh weight used for extraction. Next, the data were transformed by log2(x) and normalized by subtracting the mean of the abundances of all metabolites in the sample and submitted to principal component analysis (PCA) and ANOVA test (*P* < 0.05). Metabolites that showed statistical significance in the ANOVA test had the relative abundance profiles represented by heat map with hierarchical clustering, with the abundances previously standardized by *Z*‐score. Furthermore, *t*‐test was used to compare the means of the mutants than Col‐0 in each CO_2_ condition. GC‐MS and LC‐MS data processing was performed using Perseus 1.6.15 (Tyanova *et al*., 2016) and MetaboAnalyst 5 (Pang *et al*., 2022). Correlation and regression analyses were carried out using the software's Metabolanalyst and Sigma Plot®, respectively. The parameters significantly correlated with *g*_S_ (*P* < 0.05) were displayed in a network build using Cytoscape®.

## Results

### Assessment of NTRA/B‐TRXo1 system under ambient CO_2_ conditions

**Fig. 1:**
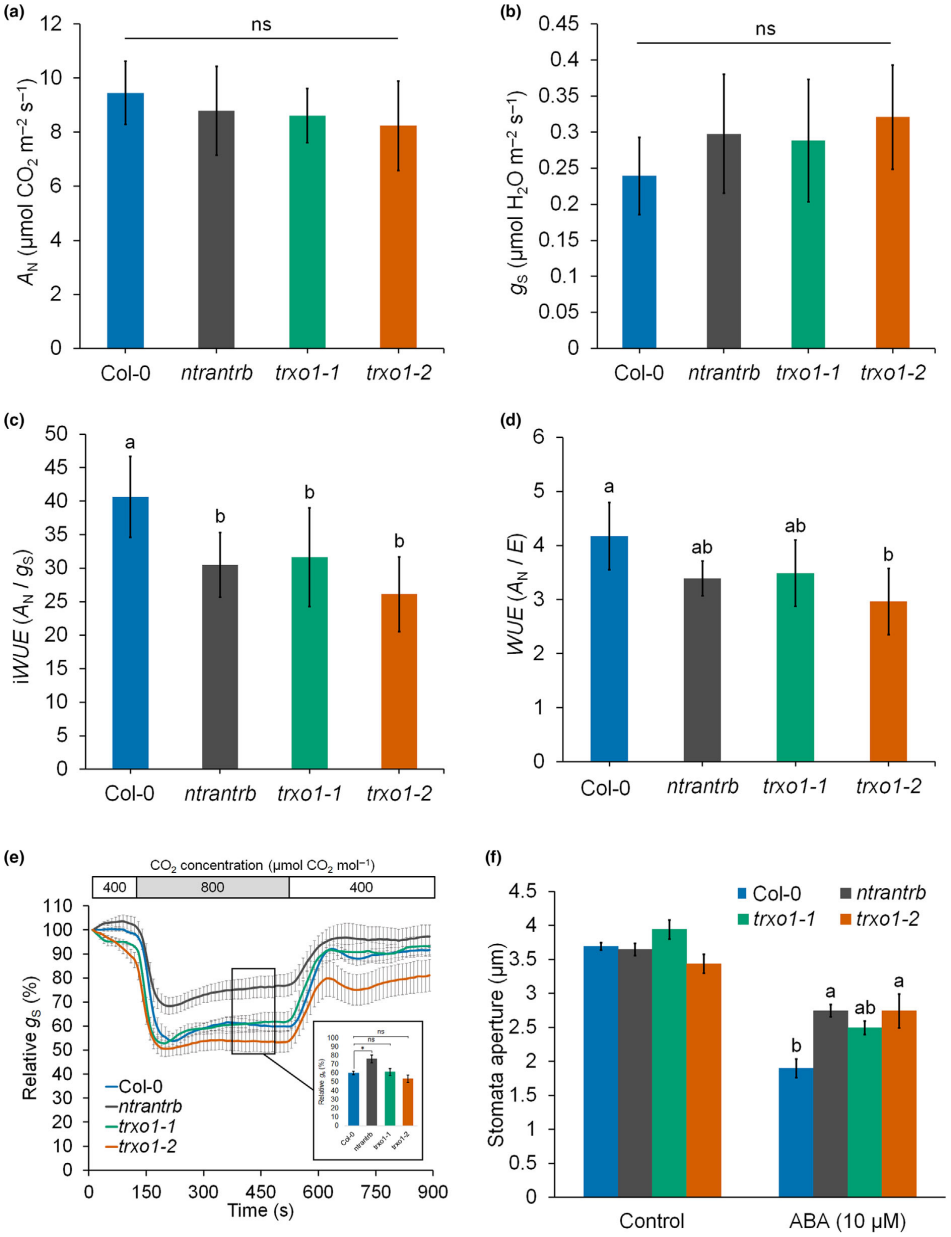
Gas exchange parameters and stomatal dynamics in *Arabidopsis* Col‐0 and *ntrantrb*, *trxo1‐1*, and *trxo1‐2* mutant plants grown under standard conditions. (a) Net photosynthesis– (*A*_N_). (b) Stomatal conductance (*g*_S_). (c) Intrinsic water‐use efficiency (iWUE). (d) Instantaneous WUE. (e) Stomatal conductance (*g*_S_) dynamics during step changes in CO_2_ concentration (400→800→400 ppm), showing stomatal closure under elevated CO_2_ and reopening after the return to ambient CO_2_. The data points were collected at 10s intervals and are presented as mean ± SE. (f) Stomatal aperture in response to the application of exogenous abscisic acid (10 μM). Data represent mean ± SE (*n* = 5). Different letters above the bars indicate significant differences (*P* < 0.05) according to Tukey's test. ns, not significant.

As a preliminary approach, plants were grown under ambient CO_2_ conditions to establish a physiological baseline in the absence of external stressors and to identify potential roles of the NTRA/B‐TRXo1 system on the regulation of photosynthesis and stomatal movements. Net photosynthetic rates (*A*_N_; Fig. 1a) and stomatal conductance (*g*_S_; Fig. 1b) did not differ significantly between Col‐0 and the *ntrantrb* or *trxo1* mutant lines, indicating that these mutations do not alter net CO_2_ assimilation or steady‐state *g*_S_ under ambient CO_2_ conditions. However, intrinsic WUE (iWUE; *A*_N_/*g*_S_) was significantly lower in all mutant lines, while the instantaneous WUE (*A*_N_/*E*) was reduced only in *trxo1‐2* line as compared with Col‐0 plants (Fig. 1c,d).

To investigate whether the lack of extraplastidial NTR/TRXs affects stomatal responsiveness to CO_2_, we monitored the dynamic changes in *g*_S_ during a stepwise change in CO_2_ concentration. As expected, *g*_S_ decreased over time in response to elevated CO_2_ (800 ppm), but this effect was notably attenuated in *ntrantrb* plants (Fig. 1e). When CO_2_ concentration was subsequently reduced from 800 to 400 ppm, *g*_S_ increased again, reflecting stomatal reopening. The steady state of the *g*_S_ at 800 ppm of CO_2_ of the *ntrantrb* double mutant was *c*. 75% of the *g*_S_ observed in this mutant under ambient CO_2_ (400 ppm of CO_2_), in contrast to the other genotypes, which maintained values between 50 and 60% (Fig. 1e). We also examined stomatal aperture in response to exogenous ABA. Under control conditions (no ABA application), stomatal aperture did not differ significantly among the genotypes (Fig. 1f). Upon application of 10 μM ABA, all genotypes showed decreased stomatal aperture, but a prominent effect on the WT was observed. While Col‐0 showed a *c*. 50% reduction, all mutant lines exhibited lower sensitivity to ABA treatment, with a reduction of only *c*. 30%. Taken together, these results suggest that redox processes regulated by NTRA/B enhance stomatal responsiveness to CO_2_ and ABA, while TRXo1 improve stomatal sensitivity to ABA but not to eCO_2_.

### The *ntrantrb* and *trxo1* genotypes exhibit altered *g*_S_ and iWUE responses under eCO_2_ conditions

**Fig. 2:**
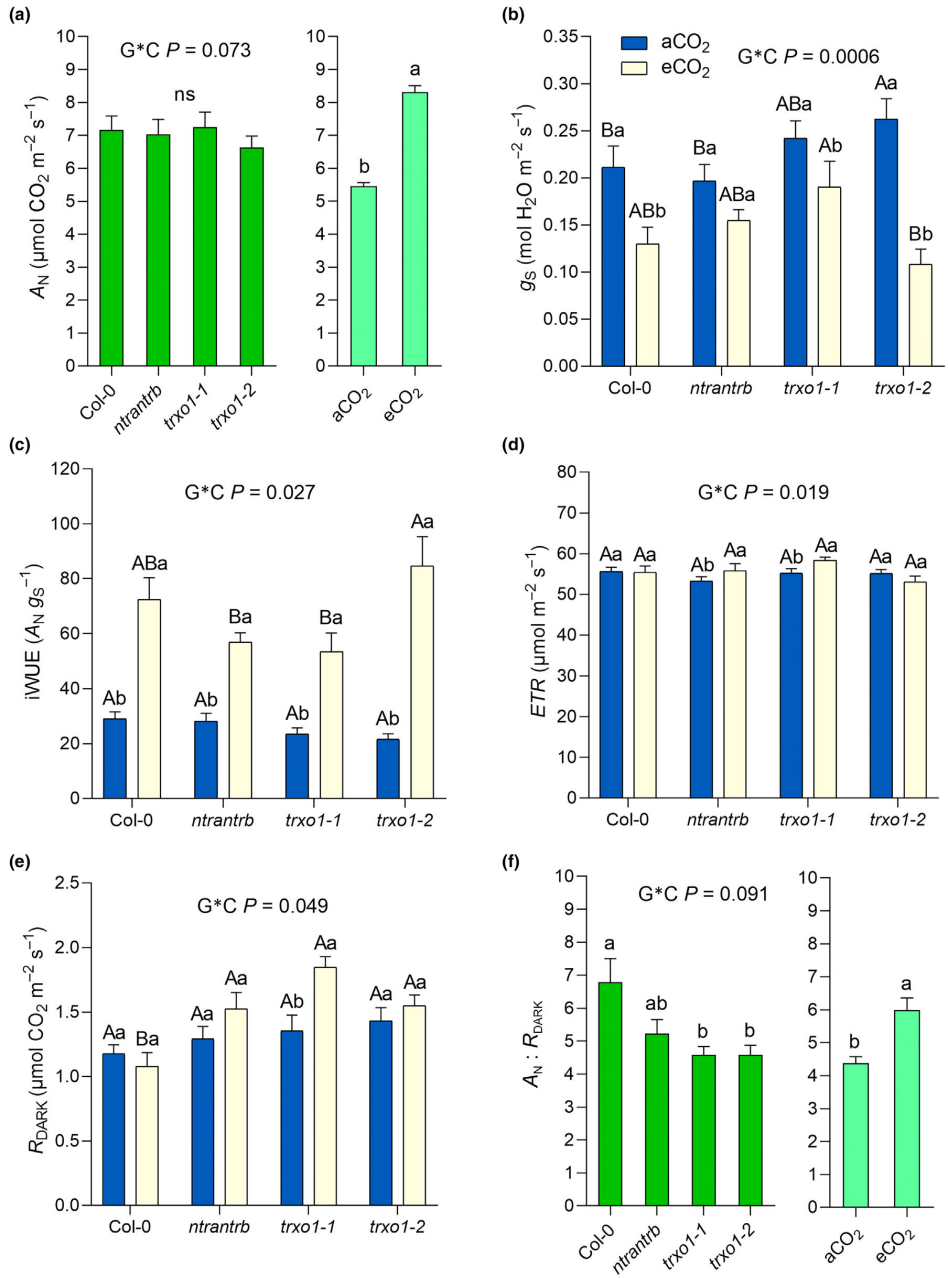
Gas exchange parameters in *Arabidopsis thaliana* Col‐0 and *ntrantrb*, *trxo1‐1*, and *trxo1‐2* mutant plants grown under ambient (aCO_2_) and elevated (eCO_2_) CO_2_ conditions. (a) Net photosynthesis (*A*_N_). (b) Stomatal conductance (*g*_S_). (c) Intrisic water‐use efficiency (iWUE). (d) Electron transport rate. (e) Nocturnal respiration (*R*_DARK_). (f) *A*_N_ : *R*_DARK_ ratio. Data represent mean ± SE of the mean (*n* ≥ 6). Uppercase letters compare different genotypes within the same condition, whereas lowercase letters compare the same genotype across CO_2_ conditions. Means were compared using Tukey's test at 5% probability. G*C indicates the genotype × CO_2_ interaction. When this interaction was not significant (*P* > 0.05), factors were analyzed separately. ns, not significant.

Given the altered stomatal responses to CO_2_ (Fig. 1e) and ABA (Fig. 1f) observed in NTRA/B and TRXo1‐deficient plants, we hypothesized that these mutants would display distinct gas exchange patterns when grown under eCO_2_. The WT and the mutants were grown under both aCO_2_ and eCO_2_ conditions. The interaction between genotypes and CO_2_ concentrations was not significant for *A*_N_; but increases were observed in all genotypes solely as a function of eCO_2_ (Fig. 2a). The *g*_S_ decreased significantly in all genotypes under eCO_2_, except for the *ntrantrb* double mutant (Fig. 2b). Under aCO_2_, *g*_S_ in *trxo1‐2* line was significantly higher than in Col‐0; however, it exhibited a strong decrease under eCO_2_. While the *g*_S_ decreased by 33% in Col‐0, it was 63% in *trxo1‐2* mutant. These results indicate that the lack of NTRA/B hampers *Arabidopsis* stomata to acclimate to long‐term eCO_2_.

The iWUE increased in all genotypes under eCO_2_ (Fig. 2c). However, *ntrantrb* and *trxo1‐1* mutant plants exhibited lower iWUE than Col‐0, suggesting impaired optimization of iWUE when grown under eCO_2_. By contrast, the *trxo1‐2* mutant exhibited a significant increase in iWUE, consistent with its lower *g*_S_ in this line under eCO_2_ (Fig. 2c). Differences between the *trxo1* mutant alleles for certain physiological and metabolic parameters have also been reported in previous studies (Calderón *et al*., 2018; Sánchez‐Guerrero *et al*., 2019), suggesting allele‐specific effects and possible compensation by other components of the thiol‐redox network. Slight increases in the electron transport rate were observed in *ntrantrb* and *trxo1‐1* mutants, but not in the WT and the *trxo1‐2* line, in response to eCO_2_ (Fig. 2d). Dark respiration (*R*_dark_) was higher in all mutants than in the WT under eCO_2_, whereas no differences among the genotypes were observed under aCO_2_ (Fig. 2e). We observed a general increase in *A*_N_/*R*_DARK_, all genotypes as a function of eCO_2_; however, all mutants, especially *trxo1* lines, showed lower *A*_N_/*R*_DARK_ than Col‐0 plants (Fig. 2f). These results suggest that the lack of extraplastidial NTR/TRXs has only a minor impact on photosynthesis, but a major effect on respiration, consistent with the established role of this system in the mitochondrial metabolism (Da Fonseca‐Pereira *et al*., 2021).

### The *ntrantrb* and *trxo1* mutations impact stomatal density and morphology

**Fig. 3:**
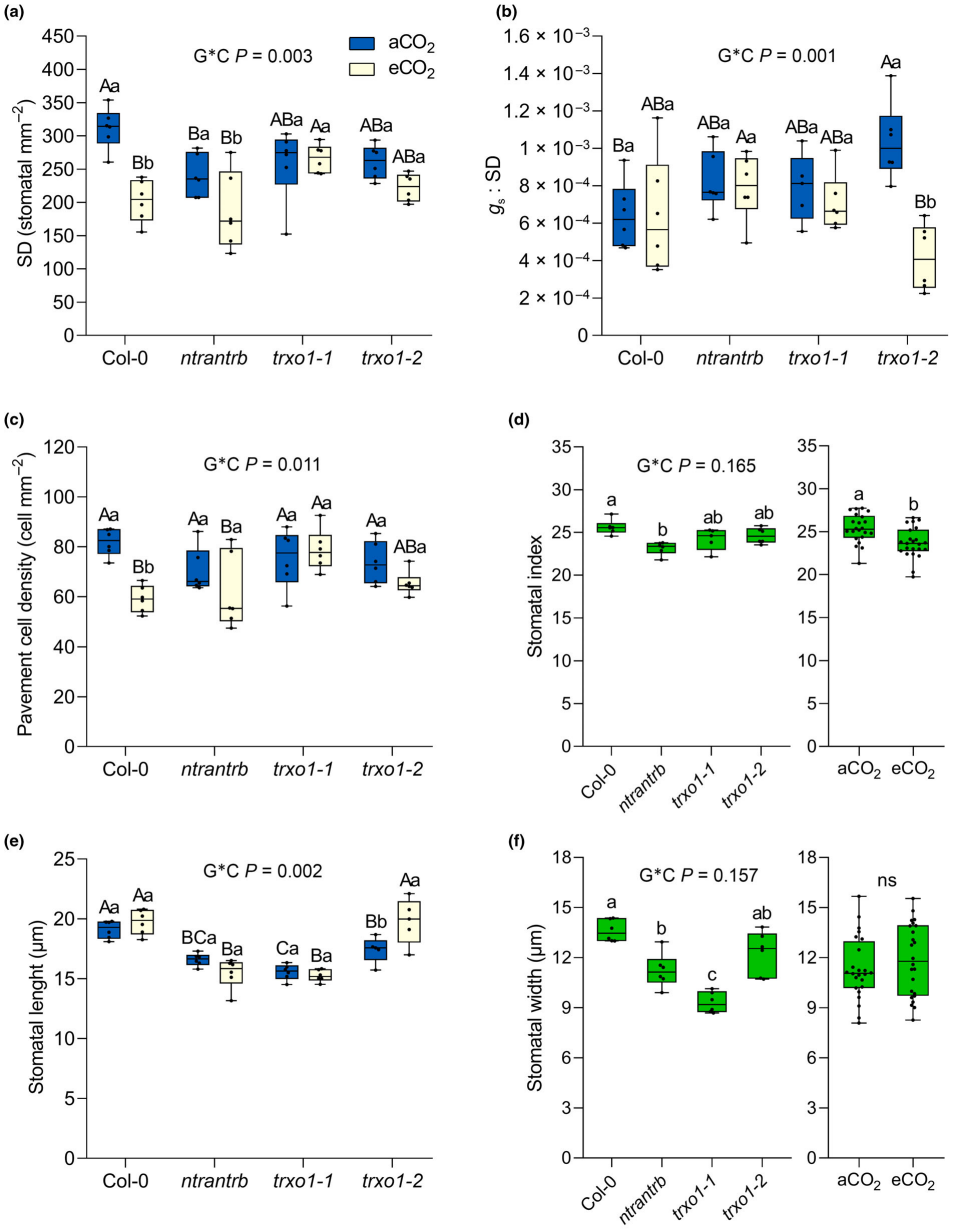
Stomatal density and morphology in *Arabidopsis thaliana* Col‐0 and in *trxo1‐1*, *trxo1‐2*, and *ntrantrb* mutant plants grown under ambient (aCO_2_) and elevated (eCO_2_) CO_2_ conditions. (a) Stomatal density (SD). (b) *g*_S_ : SD ratio. (c) Pavement cell density. (d) Stomatal index. (e) Stomatal length. (f) Stomatal width. The lower and upper boundaries of each box represent the first (Q1) and third (Q3) quartiles, respectively, while the horizontal line within the box indicates the median (*n* = 6). The whiskers extend to the minimum and maximum values within the data distribution. Uppercase letters compare different genotypes within the same CO_2_ condition, whereas lowercase letters compare the same genotype across CO_2_ conditions. Means were compared using Tukey's test at 5% probability. G*C indicates the genotype × CO_2_ interaction. When this interaction was not significant (*P* > 0.05), factors were analyzed separately. ns, not significant.

To further explore the observed differences in *g*_S_ and iWUE, we examined how the lack of NTRA/B‐TRXo1 affect stomatal density (SD) and morphology (Fig. 3). Under aCO_2_, the *ntrantrb* double mutant showed lower SD compared to Col‐0. As expected, Col‐0 plants exhibited a *c*. 30% reduction in SD under eCO_2_ (Fig. 3a). The SD also decreased in *ntrantrb* double mutant under eCO_2_. Curiously, this response was abolished in *trxo1* mutant lines (Fig. 3a). The *g*_S_ : SD ratio was significantly higher in *trxo1‐2* line than in Col‐0 under aCO_2_ (Fig. 3b). Conversely, under eCO_2_, *trxo1‐2* displayed significant decreases in this ratio, unlike the other genotypes, which remained unchanged. Pavement cell density decreased significantly only in Col‐0 under eCO_2_ (Fig. 3c). Although the stomatal index declined under eCO_2_ in all genotypes, a significant reduction was observed only in the *ntrantrb* double mutant relative to Col‐0 (Fig. 3d). Under aCO_2_, all mutants exhibited shorter stomata than Col‐0 (Fig. 3e). Under eCO_2_, stomatal length remained significantly lower in the *ntrantrb* and *trxo1‐1* lines compared with Col‐0, whereas no difference was observed between *trxo1‐2* and the WT (Fig. 3e). The values of stomatal width was reduced in all mutant lines compared with Col‐0, but this reduction was significant only in the *ntrantrb* and *trxo1‐1*, regardless of CO_2_ condition (Fig. 3f).

### CO_2_ availability differentially affects biomass accumulation in *ntrantrb* and *trxo1* mutants

**Fig. 4:**
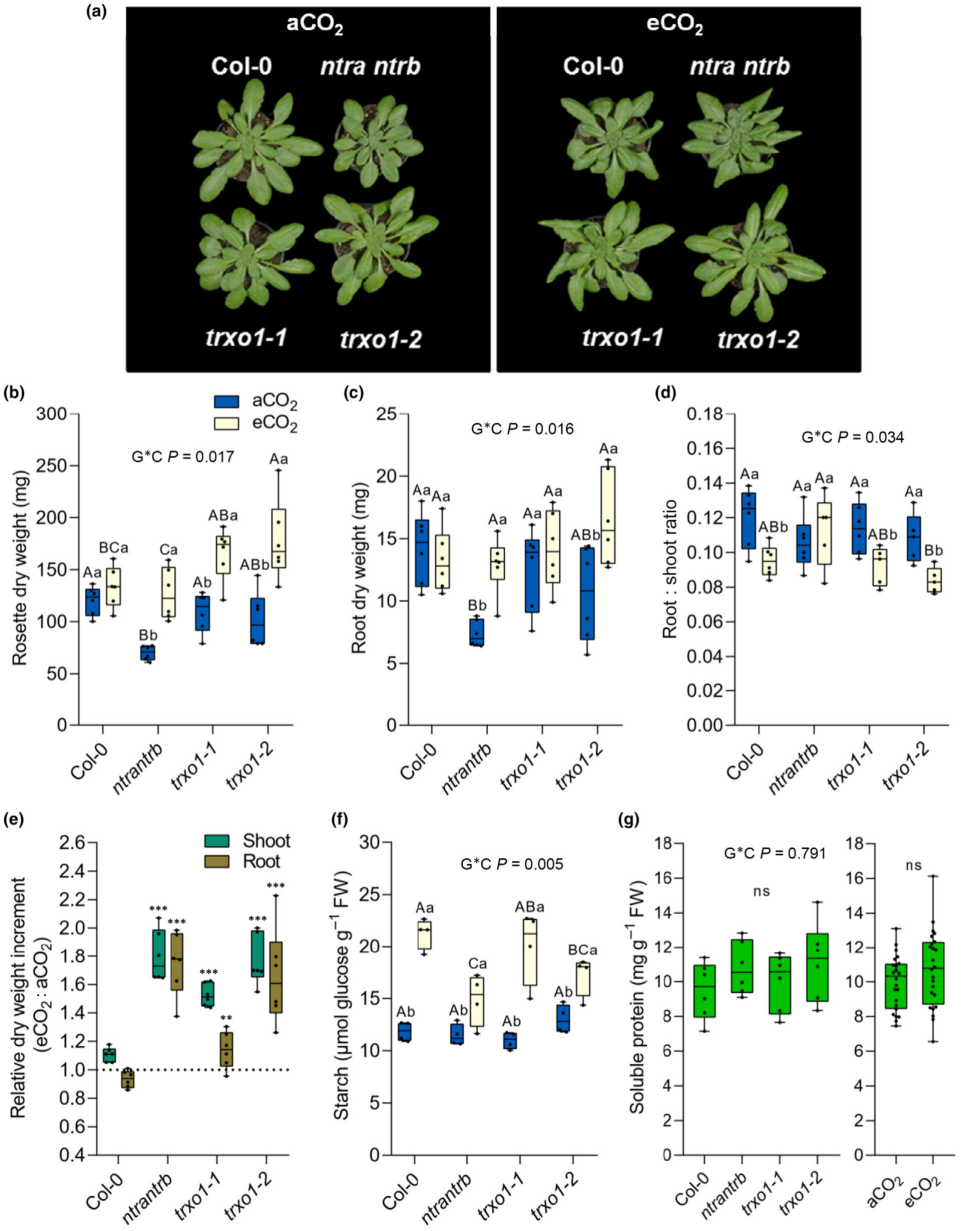
Phenotype and dry biomass accumulation in *Arabidopsis thaliana* Col‐0 and *ntrantrb*, *trxo1‐1*, and *trxo1‐2* mutant plants grown under ambient (aCO_2_) and elevated (eCO_2_) CO_2_ conditions. (a) Whole‐plant appearance of Col‐0, *ntrantrb*, *trxo1‐1*, and *trxo1‐2* under ambient (aCO_2_) and elevated (eCO_2_) CO_2_ conditions. (b) Rosette dry biomass. (c) Root dry biomass. (d) Root‐to‐shoot dry biomass ratio. (e) Relative increment in dry biomass of plants grown under eCO_2_ compared to aCO_2_. (f) Starch contents. (g) Total soluble protein contents. The lower and upper boundaries of each box represent the first (Q1) and third (Q3) quartiles, respectively, while the horizontal line within the box indicates the median (*n* = 6). The whiskers extend to the minimum and maximum values within the data distribution. Uppercase letters compare different genotypes within the same CO_2_ condition, whereas lowercase letters compare the same genotype across CO_2_ conditions. Means were compared using Tukey's test at 5% probability. G*C indicates the genotype × CO_2_ interaction. When this interaction was not significant (*P* > 0.05), factors were analyzed separately. ns, not significant. Relative biomass increment in shoots and roots was calculated as the ratio of biomass under eCO_2_ to that under aCO_2_. Mutant means were compared with the Col‐0 using Student's *t*‐test. **, *P* < 0.01; ***, *P* < 0.001.

We next assessed consequences of NTRA‐B/TRXo1 disruption on plant growth under eCO_2_ (Fig. 4). Under aCO_2_, *ntrantrb* plants appeared smaller with shorter petioles, while *trxo1* mutants resemble Col‐0. When grown under eCO_2_, *trxo1* mutants appeared visually larger than the Col‐0, while the difference in the size of the rosette between WT and *ntrantrb* disappeared (Fig. 4a). Indeed, rosette dry weight (DW) was lower in the *ntrantrb* under aCO_2_, but no difference was observed under eCO_2_ compared to the Col‐0 (Fig. 4b). Under eCO_2_, all mutants showed significantly increased rosette DW, whereas Col‐0 increased only 10% (although not statistically significant) (Fig. 4b,e). Root DW decreased significantly under aCO_2_ in *ntrantrb* double mutant and weakly in *trxo1‐2* line, but these phenotypes were reversed by eCO_2_ compared to Col‐0, which did not alter the root DW as a function of CO_2_ availability (Fig. 4c).

The root‐to‐shoot ratio showed no significant differences among genotypes under aCO_2_ (Fig. 4d). Under eCO_2_, this ratio remained statistically similar between Col‐0 and the mutants. However, only *ntrantrb* plants did not show decrease in this ratio as a function CO_2_ availability (Fig. 4d), consistent with the relative DW increment data (Fig. 4e). We also measured growth‐related parameters in the rosette. Total starch content increased in all genotypes under eCO_2_ (Fig. 4f), but the increase was smaller in *ntrantrb* (25%) compared with Col‐0 and *trxo1‐1* (*c*. 50%). Soluble protein (Fig. 4g) and Chla and *b* levels (Supporting Information Fig. S1a,b) remained unchanged across CO_2_ treatments and genotypes.

### Redox state dynamics in *ntrantrb* and *trxo1* mutants under ambient and elevated CO_2_

Levels of reduced and oxidized nicotinamide adenine dinucleotides (NADH, NAD^+^, NADPH and NADP^+^) in the genotypes studied here are shown in Fig. S2. NAD^+^ levels increased in Col‐0 and *trxo1* lines under eCO_2_, but remained unchanged in *ntrantrb* (Fig. S2a). NADH levels remained stable across all genotypes, except in *trxo1‐2*, which showed a decrease under eCO_2_ (Fig. S2b). NADP^+^ increased significantly only in *trxo1‐1* (Fig. S2c), while NADPH levels varied by genotype, with *ntrantrb* exhibiting the highest levels (Fig. S2d). The NAD^+^ : NADH ratio increased significantly under eCO_2_ across all genotypes (Fig. S2e). By contrast, the NADP^+^ : NADPH ratio was not significantly affected by eCO_2_, although *ntrantrb* plants displayed a significantly lower ratio than Col‐0 (Fig. S2f).

**Fig. 5:**
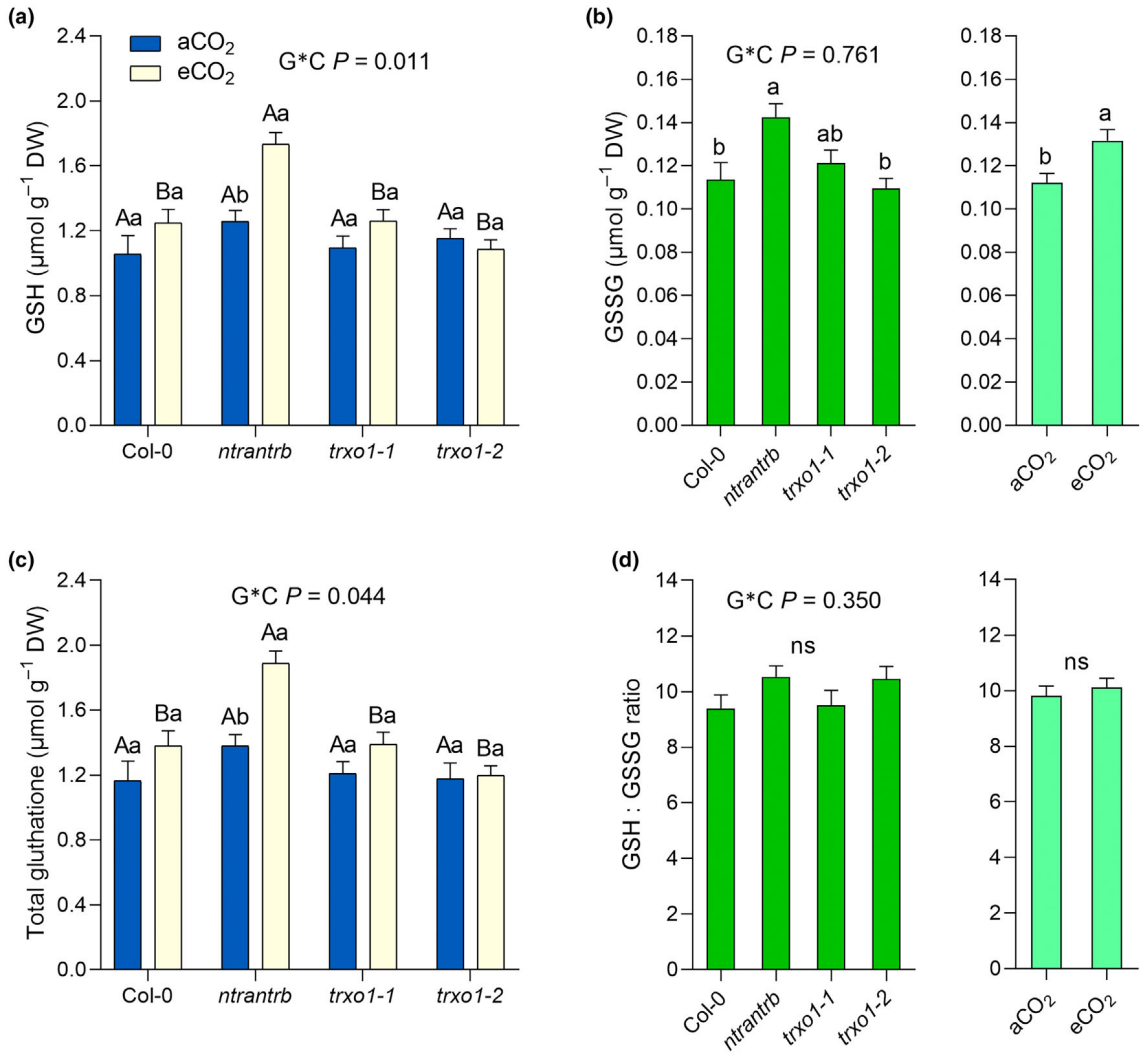
Glutathione redox status in *Arabidopsis thaliana* Col‐0 and *ntrantrb*, *trxo1‐1*, and *trxo1‐2* mutant plants grown under ambient (aCO_2_) and elevated (eCO_2_) CO_2_ conditions. (a) reduced glutathione (GSH) content. (b) oxidized glutathione (GSSG) content. (c) Total glutathione content. (d) GSH : GSSG ratio. Data represent mean ± SE of the mean (*n* = 5). Uppercase letters compare genotypes within the same CO_2_ condition, whereas lowercase letters compare the same genotype across CO_2_ conditions. Means were compared using Tukey's test at 5% probability. G*C indicates the genotype × CO_2_ interaction. When this interaction was not significant (*P* > 0.05), factors were analyzed separately. ns, not significant.

We also assessed the glutathione redox status, which plays a central role in antioxidant defense and protection against oxidative stress. Under eCO_2_, *ntrantrb* showed a significant increase in reduced glutathione (GSH) and oxidized glutathione (GSSG) levels compared with Col‐0 (Fig. 5a,b). Total glutathione was also higher in *ntrantrb* under eCO_2_ (Fig. 5c), whereas the GSH : GSSG ratio remained unchanged across genotypes and CO_2_ treatments (Fig. 5d). These results indicate that the redox metabolism is strongly affected by NTRA/NTRB mutation, given that the double *ntrantrb* mutant displayed higher levels of NAD^+^, NADP^+^, NADPH, and GSSG under aCO_2_ as well as lower NADP^+^ : NADPH ratio and higher GSH and total glutathione under eCO_2_.

### Metabolic changes in *ntrantrb* and *trxo1* mutants as a function of CO_2_ availability

**Fig. 6:**
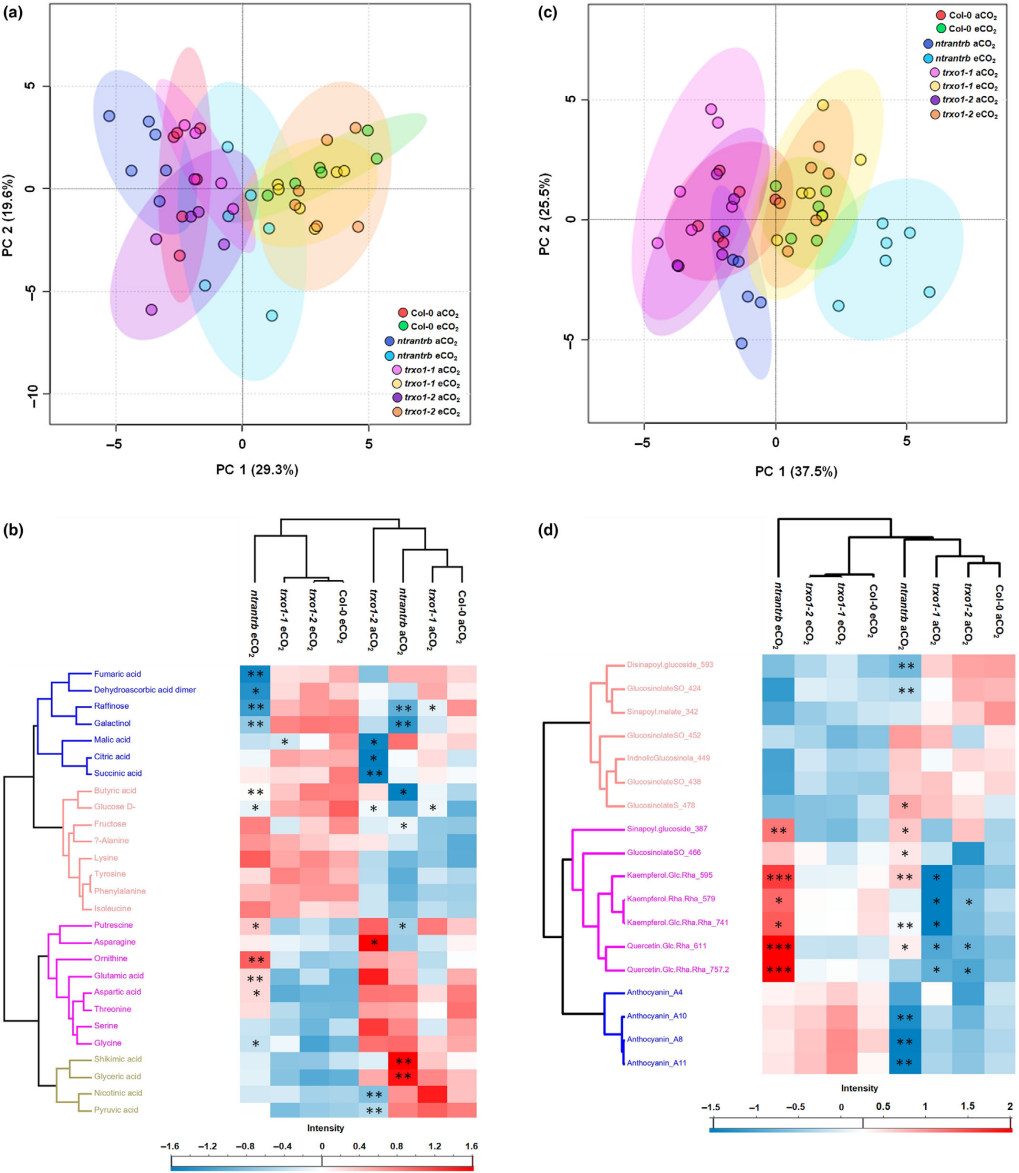
Metabolic profiling of *Arabidopsis thaliana* Col‐0 and *ntrantrb*, *trxo1‐1*, and *trxo1‐2* mutant plants grown under ambient (aCO_2_) and elevated (eCO_2_) CO_2_ conditions. (a) Principal component analysis of metabolites detected by GC‐MS and (c) LC‐MS. (b) Heat maps with hierarchical clustering showing the relative abundance of metabolites detected by GC‐MS and (d) LC‐MS. Only metabolites that showed statistical significance in the ANOVA test were included in the heat maps. Color intensity represents the relative abundance after *Z*‐score transformation. Comparison of mean (*n* = 6) was performed using Student's *t*‐test, comparing each mutant with Col‐0 within each CO_2_ condition. *, *P* < 0.05; **, *P* < 0.01; ***, *P* < 0.001.

PCA of GC‐MS dataset revealed that metabolic profiles clustered primarily according to CO_2_ availability, as evidenced by the separation of aCO_2_ and eCO_2_‐grown plants by the PC1. The only exception was the *ntrantrb* double mutant, which was positioned closer to the center of the plot (Fig. 6a).

Hierarchical clustering of GC‐MS heat map supported these findings, showing that genotypes clustered primarily as a function of CO_2_ concentration (Fig. 6b). Under aCO_2_, *trxo1‐2* was the most distinct from the other genotypes, while under eCO_2_, Col‐0 and *trxo1* mutants formed a cohesive group, with *ntrantrb* remaining distinct. Two main metabolite clusters were observed, reflecting increases or decreases in response to CO_2_ treatment. Each main clusters was divided into two subclusters based on genotype‐specific patterns (Fig. 6b). Amino acids such as lysine, tyrosine, phenylalanine, and isoleucine consistently increased under eCO_2_. Conversely, shikimic, glyceric, nicotinic, and pyruvic acids decreased in all genotypes. According to *t*‐test analysis, the *trxo1‐2* line exhibited significantly lower levels malic, succinic, and citric acids but higher levels of asparagine than Col‐0 plants under aCO_2_. On the other hand, under eCO_2_, *ntrantrb* showed reduced fumaric acid, but increased ornithine, putrescine and glutamic acid, compared to Col‐0 (Fig. 6b).

LC‐MS analysis identified secondary metabolites, which allowed a broader understanding of the role of the NTRA‐B/TRXo1 system in metabolic regulation dependent on CO_2_ availability. This analysis resembles the results of primary metabolites, in which aCO_2_ and eCO_2_‐grown plants were separated by the PC1, including the *ntrantrb* (Fig. 6c). Under aCO_2_, *ntrantrb* showed increased glycosylated flavonoids kaempferol.Glc.Rha, kaempferol.Glc.Rha.Rha, and quercetin.Glc.Rha compared to Col‐0 (Fig. 6d), consistent with the increased shikimic acid (Fig. 6b), while *trxo1‐2* showed milder effects. By contrast, under eCO_2_, Col‐0 and *trxo1* lines clustered together, with *ntrantrb* remaining dissonant (Fig. 6d). In general, eCO_2_ resulted in a decrease in glucosinolate levels and increased accumulation of flavonoid glycosides and anthocyanins. The *ntrantrb* mutant under eCO_2_ showed a substantial accumulation of kaempferol and quercetin‐derived flavonoid glycosides (Fig. 6d), being the decisive factor contributing to its unique hierarchical clustering pattern. These results indicate that the plasticity of both primary and secondary metabolism to eCO_2_ depends on NTRA/NTRB.

### Integrative analyses unveil the major determinants of the stomatal dynamics under elevated CO_2_

**Fig. 7:**
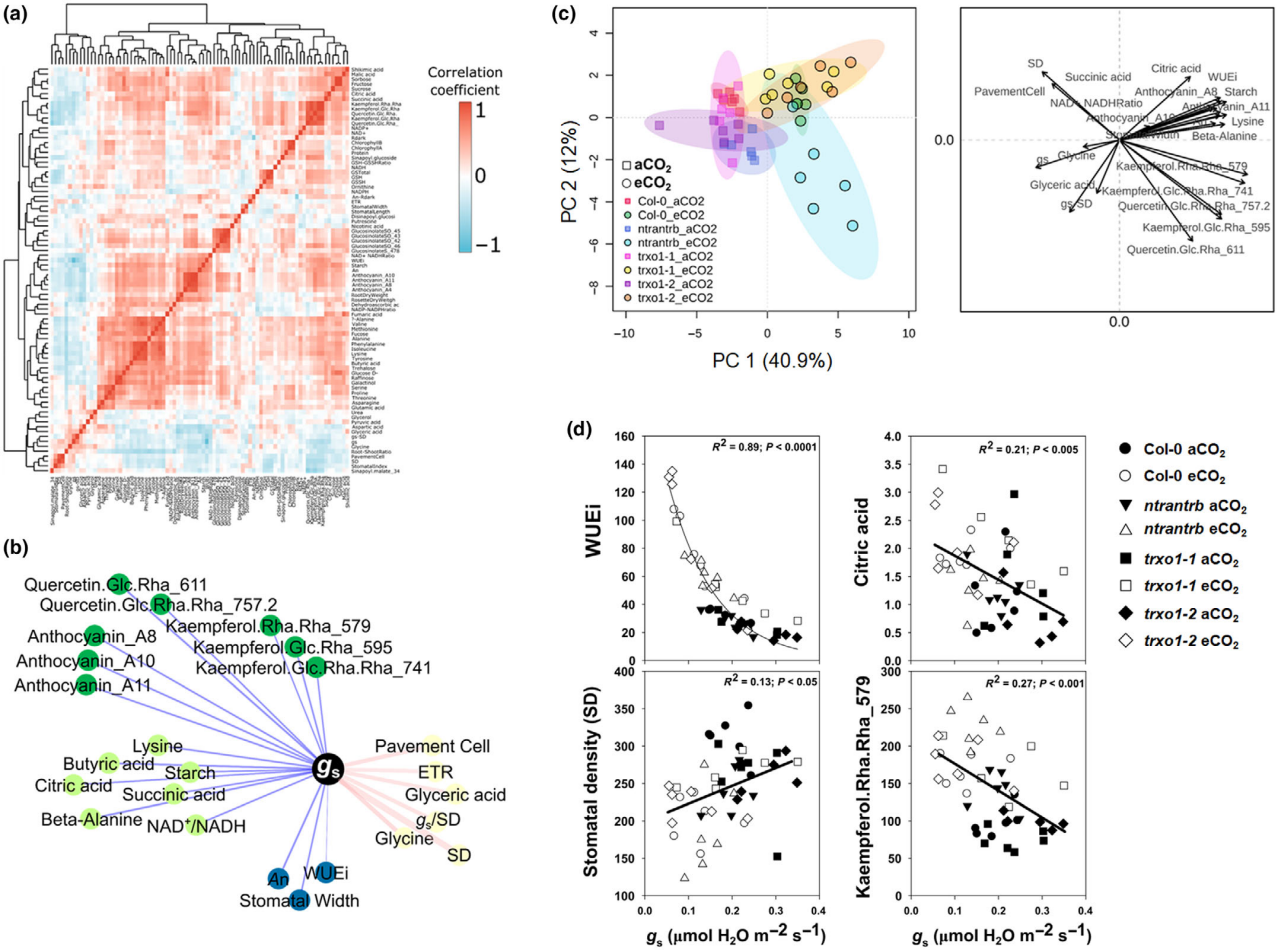
Integrative analyses identifying the major parameters and metabolites associated with stomatal conductance (*g*_s_) in *Arabidopsis thaliana* Col‐0 and the *ntrantrb*, *trxo1‐1*, and *trxo1‐2* mutant plants under ambient (aCO_2_) and elevated (eCO_2_) CO_2_ conditions. (a) Pearson's correlation matrix including all parameters measured in this study. The analysis was performed using MetaboAnalyst. (b) Correlation‐based network highlighting the 24 parameters significantly correlated with *g*_s_. Nodes represent parameters and edges represent significant correlations. Blue and red edges indicate negative and positive correlations, respectively. The network was built using Cytoscape®. (c) Principal component analysis (left) and biplot (right) based on the 24 *g*_s_‐associated parameters. Analyses were performed using MetaboAnalyst. (d) Regression analyses among selected parameters – intrinsic water‐use efficiency, stomatal density, citric acid, and kaempferol–rhamnose–rhamnose_579 – and *g*_s_. The *R* ^2^ and *P*‐value of each regression analysis are displayed in the graph. These analyses were carried out using SigmaPlot®.

We next combined all data to investigate which metabolites and stomatal and physiological parameters are mostly associated with *g*_s_ (Fig. 7a). Correlation analysis demonstrated that 24 parameters were significantly correlated with *g*_s_ (Tables S1, S2). The level of glycine and glyceric acid, *ETR*, SD, the *g*_s_ : SD ratio, and pavement cell density (cells mm^−2^) were positively correlated with *g*_s_, whereas starch, several primary, and secondary metabolites, *A*n, WUEi, stomatal width, and the NAD^+^ : NADH ratio were negatively correlated with *g*_s_ (Fig. 7b; Tables S1, S2). These correlations were highly influenced by the CO_2_ growth conditions, as evidenced by the separation of plants under aCO_2_ and eCO_2_ by the PC1 of the PCA using the 24 parameters significantly correlated with *g*_s_ (Fig. 7c). This is also evidenced in the nonlinear *g*_s_‐WUEi relationship, in which the increases in WUEi were associated with lower values of *g*_s_ and SD under eCO_2_, when compared to plants under aCO_2_ (Fig. 7d). The CO_2_ growth condition affected the level of several metabolites, helping explain both the alterations in *g*_s_ (Fig. 7d) and the PCA clustering patteen (Fig. 7c). Taken together, our results highlight that the extraplastidial TRXo1 and NTRA/B play an important role in regulating stomatal development and movement and plant acclimation to eCO_2_, likely through coordinated changes in both primary and secondary metabolisms.

## Discussion

### Disruption of the NTRA‐NTRB/TRXo1 system reduces stomatal sensitivity to CO_2_ and ABA

In the present study, we explore the hypothesis that the redox regulation of mitochondrial and cytosolic enzymatic activity plays an important role in adjusting metabolism and stomatal movements and development in response to CO_2_ concentration. This hypothesis was evaluated through the function of the NTRA, NTRB, and TRXo1 genes. Our results suggest that this system, especially NTRA/NTRB, may influence the acclimation of plants to high CO_2_.

Initially, we demonstrated that the lack of NTRA/B and TRXo1 did not alter *A*_*N*_ and *g*_S_ under aCO_2_ (Fig. 1a,b). However, clear differences emerged when stomatal dynamics were evaluated. During CO_2_ step transitions, the *ntrantrb* double mutant exhibited a diminished reduction in *g*_S_ following the increase in CO_2_ concentration from 400 to 800 ppm (Fig. 1e). Likewise, both *ntrantrb* and *trxo1‐2* demonstrated reduced sensitivity to ABA treatment (Fig. 1f). Whether these altered responses are associated with a failure in the regulatory mechanism of stomatal closure or in stomata development remains to be elucidated.

It is well‐established that stomatal closure induced by eCO_2_ or ABA occurs downstream of increased production of hydrogen peroxide (H_2_O_2_) and nitric oxide (NO) in guard cells, mediated by the activity of NADPH oxidases (RBOHs) and nitrate reductase (NR), respectively (Shi *et al*., 2015). Both RBOHs and NR depend on NADPH as a primary electron donor and are key components of the redox network regulating stomatal behavior. Given the NADPH dependence of these pathways, disruption of the NTRA/B‐TRXo1 system may indirectly influence ROS‐ and NO‐mediated signaling processes during stomatal closure. Furthermore, *ntrantrb* plants have been reported to accumulate high levels of flavonoids (Bashandy *et al*., 2009), compounds with strong antioxidant properties that can attenuate ROS accumulation and thereby weaken stomatal closure responses. This relationship between flavonoid accumulation and reduced stomatal responsiveness has been observed in several species, including *Arabidopsis*, tomato, tobacco, and apple (Watkins *et al*., 2014; Liu *et al*., 2016; Watkins *et al*., 2017; Song *et al*., 2023; Xiao *et al*., 2023). Interestingly, a similar trend has been reported in ferns, in which lower constitutive stomatal responsiveness to ABA occurs concomitantly with the greater accumulation of secondary metabolites (Cândido‐Sobrinho *et al*., 2022). Collectively, these observations support the interpretation that the lower stomatal responsiveness of *ntrantrb* to eCO_2_ and ABA could be associated to changes in secondary metabolism, particularly in antioxidant‐related compounds, which was in fact observed here (Fig. 6d) and previously reported for these mutants (Daloso *et al*., 2015; Da Fonseca‐Pereira *et al*., 2019).

It has been shown that the lack of TRXo1 increases the carbon flux through the TCA cycle (Daloso *et al*., 2015; Florez‐Sarasa *et al*., 2019; Lima *et al*., 2021; Porto *et al*., 2022). This phenotype can be attributed to the regulatory role of the TRXo1 protein on TCA cycle and associated enzymes as well as by indirect perturbations on the redox metabolism caused by the lack of NTR/TRX proteins. Specifically, TRXo1 positively regulates citrate synthase and isocitrate dehydrogenase, while negatively regulating succinate dehydrogenase and fumarase (Reinholdt *et al*., 2019; Da Fonseca‐Pereira *et al*., 2021; Timm *et al*., 2024). Such redox‐mediated control of carbon flow is essential for maintaining NAD(P)H balance and supporting alternative pathways such as AOX activity and photorespiratory adjustment through GDC regulation (Da Fonseca‐Pereira *et al*., 2021). Therefore, it seems likely that deregulated mitochondrial metabolism resulting from impairment of the NTRA/B‐TRXo1 system contributes to the phenotypes observed here, particularly given the established roles of malate and fumarate in regulating stomatal responses (Araújo *et al*., 2011; Gago *et al*., 2016; Zhang & Fernie, 2023). Moreover, the accumulation of H_2_O_2_ within mitochondrial has been implicated in ABA‐dependent stomatal signaling (Postiglione & Muday, 2023), suggesting that perturbations in the NTRA/B‐TRXo1 redox network could contribute to the reduced stomatal responsiveness observed under both CO_2_ and ABA *stimuli*. Therefore, to better understand the role of the NTRA/B‐TRXo1 system in stomatal dynamics, we evaluated in detail the stomatal and metabolic responses of these genotypes under both aCO_2_ and eCO_2_.

### The NTRA‐B and TRXo1 genes affect both stomatal traits and movements

No substantial differences in *g*_S_ were observed between the mutants and Col‐0 under aCO_2_ (Figs 1b, 2b). However, the *ntrantrb* double mutant exhibited lower values of SD, stomatal index, and stomatal length and width under aCO_2_, when compared to the WT under aCO_2_ (Fig. 3a,d–f). Punctual differences in stomatal length and width were also observed between *trxo1* lines and the WT under aCO_2_ (Fig. 3e,f). Consistent with these anatomical changes, mutant plants also displayed higher *g*_S_ : SD ratios compared to Col‐0 under aCO_2_ (Fig. 3b), especially in *trxo1* mutant lines. These findings suggest that the absence of these gene products leads to stomata that remain more open compared to Col‐0 plants under aCO_2_ conditions. This finding provides further evidence for the involvement of the NTRA/B‐TRXo1 system in the regulation of gas exchange, through as yet unknown mechanisms which likely influence both stomata development and stomatal responses to CO_2_ and ABA.

Under eCO_2_, Col‐0 plants exhibited the expected responses, reduced *g*_s_ and SD (Figs 2b, 3a), consistent with previous studies (Teng *et al*., 2006; Li *et al*., 2008). These changes were accompanied by a substantial increase in iWUE (Fig. 2c), while the *g*_s_ : SD ratio remained unchanged compared with plants grown under aCO_2_ (Fig. 3b). This pattern aligns with the view that SD can be a major determinant of *g*_S_ and WUE (Franks *et al*., 2015), although alternative interpretations have also been proposed (Flexas, 2016; Bhaskara *et al*., 2022). By contrast, mutant plants exhibited distinct responses to eCO_2_. Unexpectedly, *trxo1* mutant lines did not show a reduction in SD when grown under eCO_2_ (Fig. 3a). Instead, *g*_S_ decreased sharply (Fig. 2b), indicating a strong stomatal closure response, which resulted in a decreased in the *g*_S_ : SD ratio (Fig. 3b), a pattern not observed in the other genotypes. Conversely, the *ntrantrb* genotype resembled Col‐0 in showing reduced SD and stable of the *g*_S_ : SD values under eCO_2_ (Fig. 3a,b), but it failed to exhibit in the typical *g*_S_ reduction (Fig. 2b). These contrasting responses indicate that the absence of NTRA/B genes compromises stomatal closure under high CO_2_, while loss of TRXo1 impairs the structural adjustment of stomatal traits during CO_2_ enrichment.

**Fig. 8:**
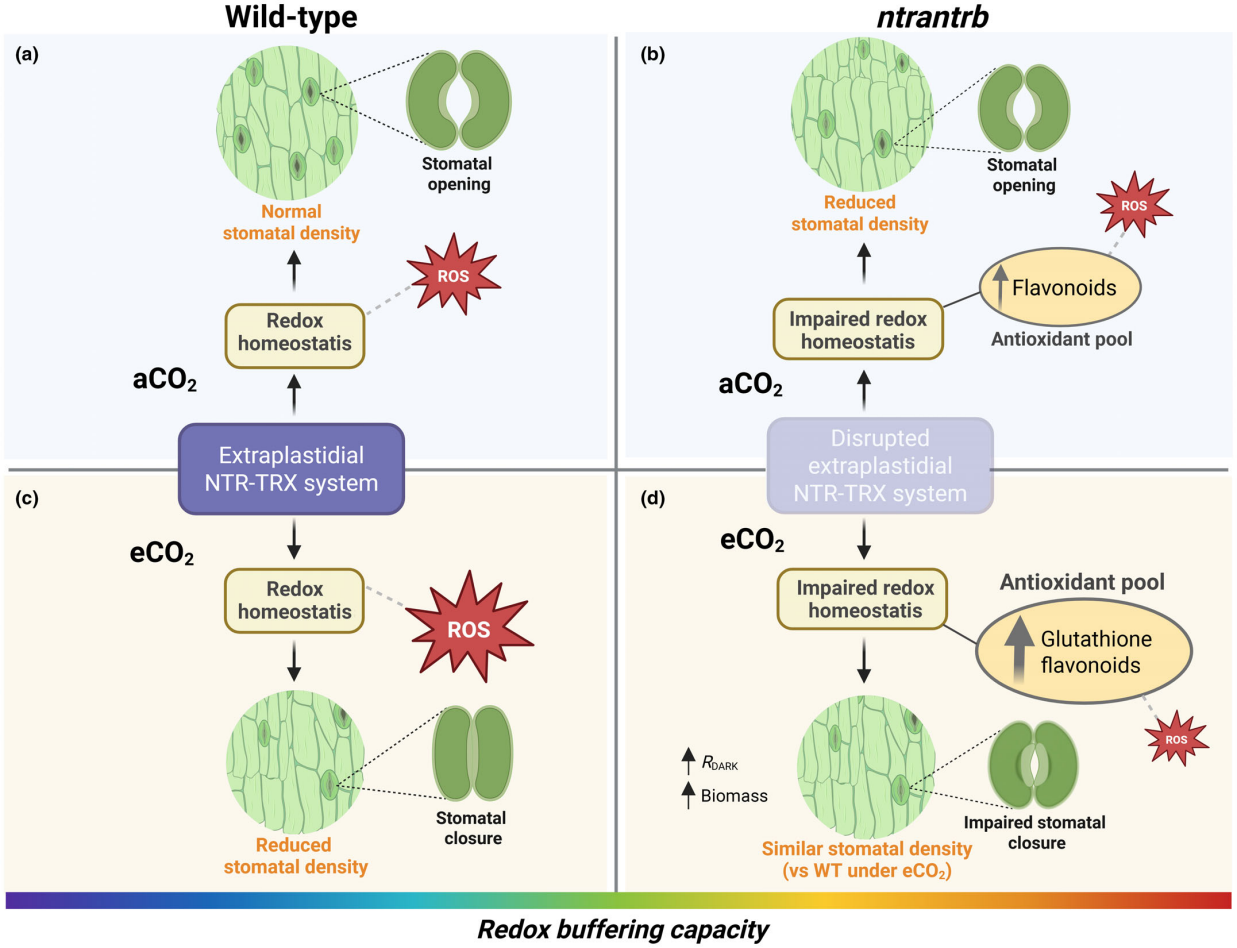
Integrative model of extraplastidial NADPH‐dependent thioredoxin reductase/thioredoxin (NTR/TRX) system‐mediated regulation of stomatal function, redox homeostasis, and metabolic responses under ambient and elevated CO_2_ in *Arabidopsis thaliana*. (a) Under ambient CO_2_ (aCO_2_), wild‐type (WT) (Col‐0) plants maintain balanced redox homeostasis, supporting normal stomatal density and stomatal opening. (b) By contrast, mutant plants exhibit altered redox homeostasis, associated with an increased antioxidant pool, including elevated flavonoid levels, and reduced stomatal density. (c) Under elevated CO_2_ (eCO_2_), WT plants display the expected responses, including reduced stomatal density and effective stomatal closure mediated by the abscisic acid/reactive oxygen species (ROS) signalling pathway (Chater *et al*., 2015), contributing to improved water‐use efficiency. (d) Conversely, mutant plants show impaired stomatal closure while maintaining stomatal density comparable to that of WT plants grown under the same conditions. Furthermore, mutant plants exhibit a further increase in the antioxidant pool, with higher levels of flavonoids and increased glutathione content relative to WT plants. These changes are likely associated with adjustments in ROS homeostasis, with the increased antioxidant pool, including flavonoids and glutathione, potentially contributing to the modulation of redox signaling and stomatal responses. Additionally, mutant plants exhibit increased dark respiration (*R*_dark_) and enhanced biomass accumulation under eCO_2_, indicating a broader metabolic reprogramming associated with altered redox regulation. This model summarizes the direct and indirect roles of the extraplastidial NTR/TRX system in coordinating stomatal behavior, redox balance, and metabolic responses under changing CO_2_ conditions. This figure was created in BioRender (https://BioRender.com/sgj5v0x).

The lower stomatal sensitivity to eCO_2_ observed in the *ntrantrb* genotype reflects distinct metabolic adjustments compared with the other genotypes. As noted previously, *ntrantrb* plants exhibit an exacerbated high‐flavonoid phenotype (Fig. 6d), as previously observed (Bashandy *et al*., 2009; Daloso *et al*., 2015). In addition, they accumulate higher levels of ornithine and putrescine, while fumarate levels are comparatively lower than the Col‐0 (Fig. 6b). Supporting this pattern, our integrated analysis showed that *g*_S_ correlated negatively with a group of flavonoid‐like metabolites (Fig. 7b), which also contributed strongly to the separation of the *ntrantrb* double mutant in the PCA analysis under eCO_2_ (Figs 6d, 7b,c). High flavonoid accumulation in guard cells has been negatively correlated with H_2_O_2_ levels (Watkins *et al*., 2017), and polyamines have recently been shown to antagonize ABA signaling (Liu *et al*., 2023). Both mechanisms can impair ABA or eCO_2_‐induced stomatal closure, given the convergence between the signaling pathways of these signals (Engineer *et al*., 2016). The consistent accumulation of flavonoids and glutathione‐related metabolites in *ntrantrb*, together with previous reports (Okuma *et al*., 2011; Watkins *et al*., 2017), supports the hypothesis that these compounds may contribute to the modulation of ABA‐mediated stomatal responses. Although our metabolomic approach does not resolve cell‐type‐specific accumulation, these associations provide a plausible mechanistic framework linking altered redox regulation and associated metabolic adjustments, including secondary metabolism, to stomatal function under elevated CO_2_ condition (Fig. 8). In summary, the metabolic alterations in *ntrantrb* under eCO_2_ likely restrict ROS accumulation, thereby reducing the efficiency of ABA and CO_2_‐induced stomatal closure (Chater *et al*., 2015; Singh *et al*., 2017), as well as may disrupt primary metabolism‐mediated stomatal closure mechanisms. Future studies using cell‐type‐specific approaches will be important to further disentangle these mechanisms.

### Growth and respiratory adjustments under elevated CO_2_ are modulated by the NTRA/B‐TRXo1 system

DW accumulation in roots and shoots of Col‐0 plants did not significantly change as a function of CO_2_ availability (Fig. 4b,c). Instead, starch content increased by *c*. 50% (Fig. 4f), suggesting that the additional carbon fixed under eCO_2_ was preferentially diverted toward storage rather than growth. This pattern is consistent with the stable *R*_dark_ rates observed in Col‐0 under both CO_2_ conditions (Fig. 2e) and agrees with previous observations in Arabidopsis exposed to CO_2_ enrichment (Feitosa‐Araujo *et al*., 2022). Conversely, *ntrantrb* plants exhibited severe growth limitation under aCO_2_, with *c*. 50% reductions in both root and shoot biomass, compared to the Col‐0 (Fig. 4b,c). Remarkably, this phenotype was reversed under eCO_2_, where the relative biomass increment exceeded 70% in the double mutant (Fig. 4e). Previous studies have shown that disruption of thioredoxin‐dependent redox systems can lead to substantial alterations in carbon metabolism and growth regulation in Arabidopsis under both aCO_2_ and eCO_2_ conditions (Daloso *et al*., 2015; Souza *et al*., 2023). These observations support the idea that changes in redox regulation may indirectly influence growth through metabolic reprogramming in addition to the direct effects on photosynthetic carbon assimilation. In the *trxo1* lines, a relative biomass increment was also observed under eCO_2_ (Fig. 4e), although to a lesser extent, in agreement with the increased in *R*_dark_ observed in all mutant genotypes (Fig. 2e). These results imply that the dysregulation of the redox balance caused by the lack of NTRA/B‐TRXo1 impacts the accumulation and partitioning of photoassimilates in a manner dependent on CO_2_ availability.

The effect of these mutations on NAD(P) and glutathione levels was most evident in *ntrantrb* plants, as indicated by a significantly lower NADP^+^ : NADPH ratio relative to Col‐0 (Fig. S2f) and higher glutathione concentrations under eCO_2_ (Fig. 5a–c), suggesting a substantial reconfiguration of cellular redox buffering capacity (Reichheld *et al*., 2007; Marty *et al*., 2009). Notably, levels of glutamic acid, a glutathione precursor, were also significantly higher in *ntrantrb* double mutant under eCO_2_ compared to WT (Fig. 6b). In *trxo1* plants, despite previous studies reporting an enhanced carbon flux through the TCA cycle (Florez‐Sarasa *et al*., 2019; Porto *et al*., 2022), a finding also supported by the present work (Fig. 6b), NAD(P) (Fig. S2) and glutathione levels (Fig. 5) followed patterns similar to those found in Col‐0 plants. Together, these findings indicate that disruption of NTRA/B has a stronger impact on cellular redox homeostasis than the loss of TRXo1 alone, providing a biochemical framework for the contrasting growth and stomatal phenotypes observed between these mutants.

In conclusion, our findings demonstrate that the NTRA/B‐TRXo1 system contributes to stomatal regulation and metabolic acclimation to elevated CO_2_. Both *ntrantrb* and *trxo1* mutants displayed increased biomass under eCO_2_, accompanied by increased respiratory activity and adjustments in central carbon metabolism. In *ntrantrb*, this response was accompanied by elevated flavonoid levels and broader alterations in primary and secondary metabolism, together with pronounced changes in glutathione redox homeostasis, which were associated with reduced stomatal sensitivity to eCO_2_ (Fig. 8). These results highlight the importance of extraplastidial thiol‐redox regulation in enabling gas exchange plasticity under CO_2_ enrichment. The integration of redox metabolism with stomatal behavior provides a mechanistic framework for understanding how mitochondrial and cytosolic redox networks influence whole‐plant acclimation to eCO_2_, paralleling roles previously described for NTRC (Souza *et al*., 2023). Targeting redox‐sensitive components such as TRXs and NTRs may therefore represent a potential strategy to enhance WUE and carbon assimilation under future climate atmospheric CO_2_ scenarios.

## Competing interests

None declared.

## Author contributions

AN‐N and WLA conceived the project and got funding. PF‐P performed the experiments; PF‐P, DGC, JL‐C, IK and LPS performed biochemical and physiological analyses. DFMN, RCM‐B and PFP interpreted the data and wrote the first draft of the manuscript. DMD, JG, ARF, WLA and AN‐N reviewed and contributed with the discussion of the manuscript. All authors read and approved the manuscript. PF‐P and DFMN contributed equally to this work.

## Disclaimer

The New Phytologist Foundation remains neutral with regard to jurisdictional claims in maps and in any institutional affiliations.

## Supporting information


**Fig. S1** Chl contents of *Arabidopsis thaliana* Col‐0 and *ntrantrb*, *trxo1‐1* and *trxo1‐2* mutant plants grown under ambient (aCO_2_) and elevated (eCO_2_) CO_2_ conditions.
**Fig. S2** Nicotinamide adenine dinucleotide (phosphate) levels in *Arabidopsis thaliana* Col‐0 and *ntrantrb*, *trxo1‐1*, and *trxo1‐2* mutant plants grown under ambient (aCO_2_) and elevated (eCO_2_) CO_2_ conditions.

**Table S1** Pearson's correlation matrix for all variables analyzed in this study.
**Table S2** *P*‐values associated with the correlation analysis presented in Table S1.
Please note: Wiley is not responsible for the content or functionality of any Supporting Information supplied by the authors. Any queries (other than missing material) should be directed to the *New Phytologist* Central Office.

## Data Availability

All data required to evaluate the conclusions of this study are available within the paper and its Tables S1 and S2.
