# Supplementary material for: Thioredoxin system modulates metabolic and stomatal responses to elevated CO2 in Arabidopsis
Source: New Phytol. 2026 May 29;251(4):1774–91. doi: 10.1111/nph.71302 (PMC13373855; doi:10.1111/nph.71302)
Supplement: Supplementary file 1 — Fig. S1 Chl contents of Arabidopsis thaliana Col‐0 and ntrantrb, trxo1‐1 and trxo1‐2 mutant plants grown under ambient (aCO2) and elevated (eCO2) CO2 conditions. Fig. S2 Nicotinamide adenine dinucleotide (phosphate) levels in Arabidopsis thaliana Col‐0 and ntrantrb, trxo1‐1, and trxo1‐2 mutant plants grown under ambient (aCO2) and elevated (eCO2) CO2 conditions. [file NPH-251-1774-s002.docx]

## *New Phytologist* Supporting Information

Article title: Thioredoxin system modulates metabolic and stomatal responses to elevated CO_2_ in Arabidopsis

Authors: Paula da Fonseca-Pereira^#1^, Domingos F. Mélo Neto^#1^, Rita de Cássia Monteiro-Batista^1^, Daniel Gomes Coelho^1^, Jaciara Lana-Costa^1^, Leonardo Perez de Souza^2^, Ina Krahnert^2^, Danilo M. Daloso^3^, Jorge Gago^4^, Alisdair R. Fernie^2^, Wagner L. Araújo^1^, Adriano Nunes-Nesi^*1^

Article acceptance date: 06 May 2026

The following Supporting Information is available for this article:


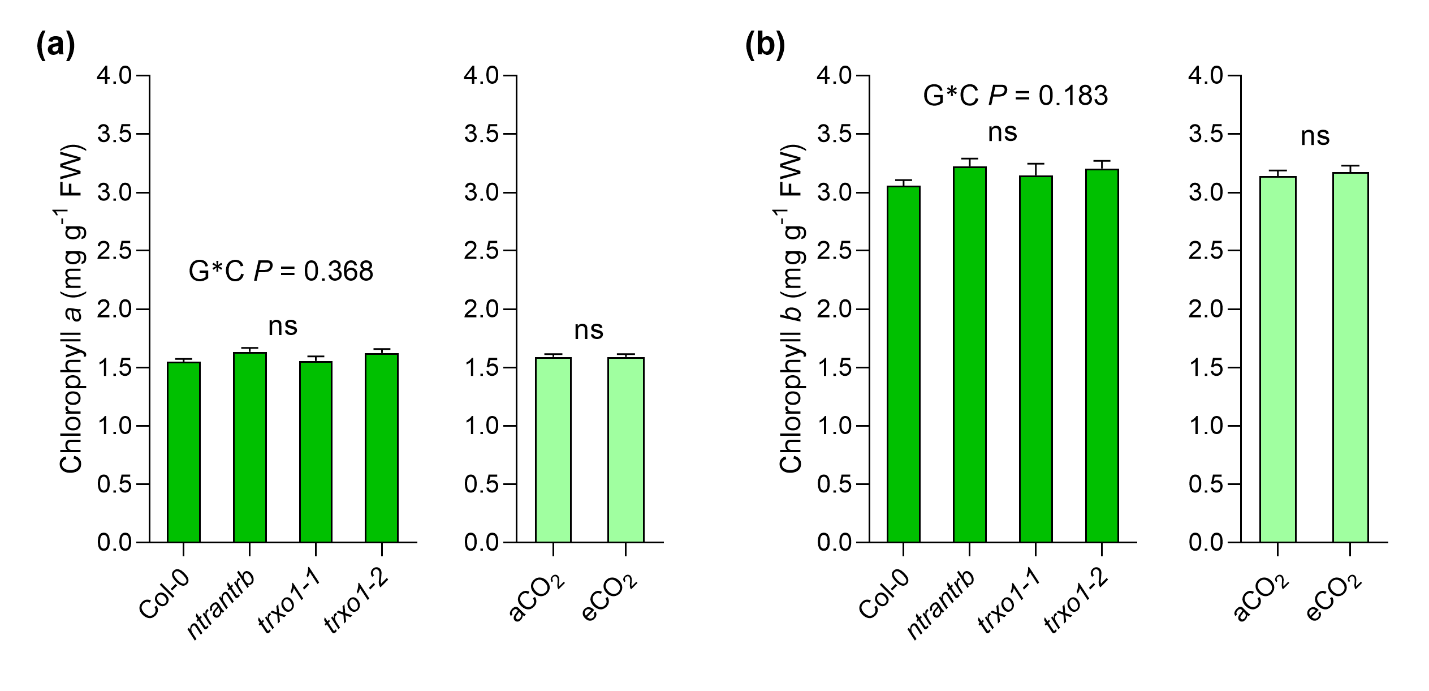
**Fig. S1** **Chlorophyll contents of *Arabidopsis thaliana* Col-0 and *ntrantrb*, *trxo1-1* and *trxo1-2* mutant plants grown under ambient (aCO_2_) and elevated (eCO_2_) CO_2_ conditions.** (a) Chlorophyll *a* content. (b) Chlorophyll *b* content. Data represent mean ± standard error of the mean (n=5). G * C indicates the genotype x CO_2_ interaction. When this interaction was not significant (*p*>0.05), factors were analyzed separately. ns = not significant.

**
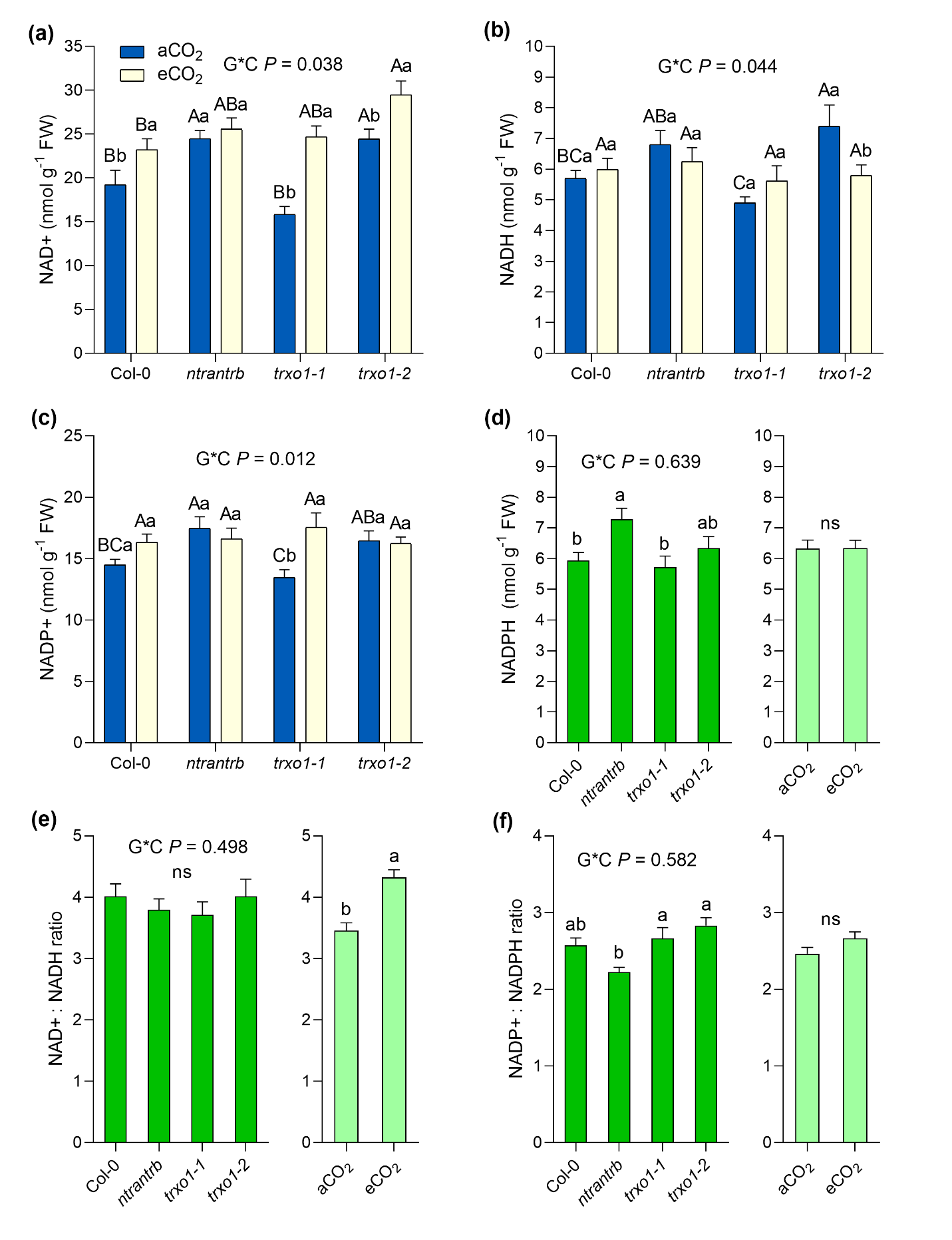
**

**Fig. S2 Nicotinamide adenine dinucleotide (phosphate) levels in *Arabidopsis thaliana* Col-0 and *ntrantrb*, *trxo1-1* and *trxo1-2* mutant plants grown under ambient (aCO_2_) and elevated (eCO_2_) CO_2_ conditions.** (a) NAD^+^ content. (b) NADH content. (c) NADP^+^ content. (d) NADPH content. (e) NAD^+^ to NADH ratio. (f) NADP^+^ to NADPH ratio. Data represent mean ± standard error of the mean (n=5). Uppercase letters compare genotypes within the same CO_2_ condition, whereas lowercase letters compare the same genotype across CO_2_ conditions. Means were compared using Tukey’s test at 5% probability. G * C indicates the genotype x CO_2_ interaction. When this interaction was not significant (*p*>0.05), factors were analyzed separately. ns = not significant.
